# Effects of repeated application of sulfadiazine-contaminated pig manure on the abundance and diversity of ammonia and nitrite oxidizers in the root-rhizosphere complex of pasture plants under field conditions

**DOI:** 10.3389/fmicb.2013.00022

**Published:** 2013-02-14

**Authors:** Julien Ollivier, Daniela Schacht, Reimo Kindler, Joost Groeneweg, Marion Engel, Berndt-Michael Wilke, Kristina Kleineidam, Michael Schloter

**Affiliations:** ^1^Institute of Soil Ecology, Technical University MunichNeuherberg, Germany; ^2^Research Unit Environmental Genomics, Helmholtz Zentrum MünchenNeuherberg, Germany; ^3^Institute of Ecology, Technical University BerlinBerlin, Germany; ^4^Agrosphere Institute, Forschungszentrum JülichJülich, Germany

**Keywords:** sulfadiazine, rhizosphere, ammonia-oxidizing microbes, nitrite-oxidizing bacteria, nitrification

## Abstract

In a field experiment, the impact of repeated application of the antibiotic sulfadiazine (SDZ)-contaminated pig manure was assessed on functional microbial communities involved in ammonia and nitrite oxidation in the root-rhizosphere complexes (RRCs) of diverse plants composing a pasture. We surveyed the abundance of ammonia-oxidizing archaea (AOA) and bacteria (AOB) as well as *Nitrobacter*- and *Nitrospira*-like nitrite-oxidizing bacteria (NOB) by quantitative PCR (qPCR), and the diversity of *amoA* AOA and *Nitrobacter*-like *nxrA* amplicons using a cloning-sequencing approach. Whereas the first SDZ-contaminated manure application caused only slight effects on the investigated microbial communities and did not change the diversity and abundance pattern significantly, the second application of SDZ-contaminated manure induced an up to 15-fold increased ratio of AOA:AOB and a reduction of *nrxA* genes. The diversity of AOA *amoA* increased after the second application of SDZ-contaminated manure compared to the control treatment whereas a clear reduction of *nrxA* OTUs was visible in the same samples. The results indicate that the application of SDZ may principally affect nitrite oxidation by NOB and alternative pathways like nitrite reduction might be favored under these conditions.

## Introduction

Nitrification rates in soils can be considered as an important indicator for sustainable use. Ammonium derived from ammonia is of high importance as the base product of this process, for plant nutrition and biomass formation. Catabolism and leaching of the end product, nitrate, often causes significant losses of nitrogen (N) as well as significant environmental problems such as contamination of groundwater by leaching, or the formation of the greenhouse gas N_2_O by denitrifying microbes (Ollivier et al., 2011). Nitrification is a two-step processes including (i) the oxidation of NH^+^_4_ to NO^−^_2_ via hydroxylamine by ammonia-oxidizing microbes (Kowalchuk and Stephen, 2001; Leininger et al., 2006) and (ii) the oxidation of NO^−^_2_ to NO^−^_3_ by nitrite-oxidizing bacteria (NOB) (Prosser, 1989). Ammonia oxidation and nitrite oxidation are both performed by phylogenetically well separated microorganisms. Ammonia oxidation is performed by autotrophic bacteria belonging to two specific groups of β- and γ-proteobacteria (AOB; Bock and Wagner, 2006) and probably mixotrophic archaea (AOA) recently assigned to the phylum Thaumarchaeota (Spang et al., 2010); NOB are broadly distributed among the α-, β-, γ-, and δ-proteobacteria as well as the *Nitrospira* phylum (Spieck and Bock, 2005). So far, no organism capable of performing both the oxidation of ammonia and the oxidation of nitrite has been identified (Schramm et al., 1996; Gieseke et al., 2003).

Despite the fact that an efficient nitrification requires the presence of both ammonia oxidizers and nitrite oxidizers, most studies in have focused on factors driving abundance, diversity and activity of ammonia oxidizers. ISI Web of Knowledge (http://www.webofknowledge.com/) reveals almost 2500 articles using the keywords “ammonia oxidation” and “soil,” whereas only 600 hits were found using “nitrite oxidation” and “soil.” Less than 30 peer-reviewed studies investigated both processes. The reason for this strong focus on ammonia oxidation is mainly related to several studies from the last century where the oxidation of ammonia has been considered as rate limiting for the whole process of nitrification (Prosser, 1989). Main findings from that time include varying copy numbers of AOB (Phillips et al., 2000) and nitrite concentrations below the detection limit in many soil samples (Burns et al., 1995), indicating that once nitrite is formed it can be quickly oxidized to nitrate. During that time, the existence of ammonia-oxidizing archaea (AOA) was not proven and ammonia oxidation in soil was thought related to some proteobacteria.

With the detection of AOA, the paradigm of nitrification changed and new questions have been raised concerning the role of AOA for nitrification, including (i) the transformation of hydroxylamine (NH_2_OH) to nitrite by AOA, since no homolog of bacterial HAO gene (*hao*) encoding the enzyme catalyzing the oxidation of NH_2_OH to NO^−^_2_ has been found in the genome of AOA (ii) the functional role of the described *nirK* sequences (encoding a putative nitrite reductase) from AOA (Bartossek et al., 2010) (iii) possible pathways indicating mixotrophy of AOA, and (iv) the functional interplay between AOA and NOB populations. Differences between AOA and AOB may result in dissimilarities in the response of these communities to inhibitive agents (Schauss et al., 2009). Therefore, the aim of this study was to investigate the response of ammonia (AOA and AOB) and nitrite oxidizers (NOB) to a repeated application of antibiotics (sulfadiazine; SDZ) in the root rhizosphere complex (RRC) of a mixture of typical grassland plant species at different plant development stages during the growing season in a field study, and thus to link the data to ammonium and nitrate fluxes in soil. In order to assess changes in the genetic potential, we analyzed the abundance pattern of genes encoding key enzymes of ammonia oxidation (*amoA* encoding the ammonia monooxygenase) in both AOB and AOA, and nitrite oxidation in *Nitrobacter*-like NOB (*nxrA* encoding the nitrite oxidoreductase). *Nitrospira*-like NOB were quantified based on analysis of 16S rRNA genes. Moreover, the functional diversity of archaeal *amoA* and *Nitrobacter*-like *nxrA* was determined using a cloning-sequencing approach.

As the administration of antibiotics to treat infectious diseases is a common practice in animal husbandry, substances like the sulfonamide, which is mainly used in pig production (Burkhardt et al., 2005), are poorly adsorbed by the animal and excreted mostly unaltered in urine and feces together with various metabolites (Elmund et al., 1971; Alcock et al., 1999; Halling-Sørensen, 2001; Lamshöft et al., 2007), and thus reach the soil ecosystem via manuring. By inhibiting folic acid metabolism, SDZ impairs growth of most Gram-positive and many Gram-negative bacteria (Brown, 1962). The occurrence of SDZ in soil might therefore alter the microbial community structure as well as its activity, and modify kinetics of important turnover processes such as nitrogen (N) cycling. We hypothesized that SDZ mainly affects the ratio of archaeal vs. bacterial ammonia oxidizers, whereas *Nitrospira*- and *Nitrobacter-like* nitrite oxidizers are both inhibited by the application of the contaminated manure, despite their different phylogenetic classification. Therefore, under antibiotic pressure, we postulate that nitrite oxidation might be the rate limiting step of nitrification.

## Materials and methods

### Experimental design

An agricultural field located near Merzenhausen, Germany (50° 56′ 3″ N, 6° 17′ 31″ E) which only received inorganic fertilizer within the last 30 years and therefore has not been in contact with manure derived antibiotics during this period, was chosen as experimental site. The management included a cropping sequence consisting of potatoes, wheat, barley, sugar beets, and pasture The soil has been characterized as a silt loam (pH 7.2, Orthic Luvisol; Table 1). The experiment was setup as a randomized split plot design (plots size: 3 × 6 m) with a mixture of pasture plants (47% *Lolium perenne*, 17*% Phleum pratense*, 20% *Festuca pratensis*, 10% *Poa pratensis*, and 6% *Trifolium repens*) using manure from untreated pigs (PM) and SDZ-treated pigs (PMSDZ), respectively, with four replicates for each variant providing height plots in total.

**Table 1:** **Physical characterization of the soil used in the experiment**.

**Mean proportion (%)**	
Clay	15.4
Silt	78.2
Sand	6.4
WHC_max_	45.8
C_org_	1.2

Manure was applied twice during the vegetation season (applied total N ranged from 16 to 19 g N m^−2^ and from 3 to 6 g N m^−2^, respectively; Table 2). The first manure application (30 m^3^ ha^−1^) was completed in May 2009. The second application (10 m^3^ ha^−1^) occurred 48 d later. Pasture plots were cut one day prior to the second manure application. The amount of SDZ applied in treatments with PMSDZ was equivalent for each of the two applications to 100 mg SDZ m^−2^. The amount of the CaCl_2_-extractable SDZ recovered from soil during the experimental period declined quickly after each manure application (Rosendahl et al., 2011).

**Table 2:** **Chemical characterization of the pig manure (PM) and SDZ-contaminated pig manure (PMSDZ) applied in the experiment**.

**Manure**	**pH**	Total N^*^	NH_4_-N^*^	P_2_O_5_^*^	K_2_O^*^	MgO^*^	CaO^*^
**FIRST APPLICATION**							
PM	8.0	6.19	3.66	4.21	3.77	2.57	2.89
PMSDZ	7.6	5.43	4.46	3.35	3.30	1.96	2.87
**SECOND APPLICATION**							
PM	8.7	5.50	3.13	4.02	3.90	2.61	2.88
PMSDZ	8.3	3.47	2.39	1.05	3.54	0.72	1.16

* *kg/m^3^*.

Rhizosphere samples were collected from all plots at day 1 (after the first application of the manure) 7, 14, and 42 d; 49, 56, 63, and 106 d (1, 8, 15, and 58 d after the second manure application, respectively). For each plot, 10 subsamples were randomly collected, mixed, and homogenized to obtain one composite sample per plot. Composite samples taken from plots with the same treatments were considered replicates. After shaking the roots vigorously, the RRC samples (roots and adhering soil) were divided equally into two sub-samples. One part was immediately shock-frozen in liquid nitrogen and stored at −80°C prior to DNA extraction; the other part was directly extracted with 0.01 M CaCl_2_ for determination of ammonium-N (NH^+^_4_-N) and nitrate-N (NO^−^_3_-N) concentrations.

### Inorganic nitrogen fraction in the RRC

RRC (300 mg) was agitated using an overhead shaker for 30 min with 5 ml of 0.01 M CaCl_2_. After filtration, ammonium-N and nitrate-N measurements were performed on Nanocolor 300D photometer from Macherey Nagel (Germany) by using the Nanocolor Ammonium 3 kit and the Nanocolor nitrate 50 kit, respectively (Macherey Nagel, Germany), according to the manufacturer's suggested protocols.

### Nucleic acid extraction

RRC DNA was directly extracted after a bead beater lysis step (Bertin Technologie, France), using the FastDNA SPIN kit for soil according to the manufacturer's suggested protocol (MP biomedicals, Germany). Quality and quantity of the extracted DNA were checked with a spectrophotometer (Nanodrop, PeqLab, Germany).

### Abundance of functional genes

Quantitative PCR (qPCR) of genes encoding key enzymes of ammonia oxidation (*amoA* encoding the ammonia monooxygenase) in both ammonia-oxidizing bacteria (AOB) and AOA, and nitrite oxidation in *Nitrobacter*-like NOB (*nxrA* encoding the nitrite oxidoreductase) was used to determine the density of the functional communities involved in nitrification. In addition, the abundance of *Nitrospira*-like NOB was quantified using the 16S rRNA *Nitrospira* gene as target since no primers targeting *Nitrospira*-like *nxrA* were available (Wertz et al., 2012). An absolute quantification of all investigated target genes using a SYBR® Green I-based detection (Applied Biosystems, Germany) was carried out in 25 μL in triplicates on the ABI Prism 7300 Cycler (Applied Biosystems). The reaction mixture consisted of 15 μg bovine serum albumin (Sigma-Aldrich, Germany), 0.2 μM of each primer for *amoA* AOA, *nxrA*, and 16S rRNA *Nitrospira* gene amplification and 0.3 μM of each primer for *amoA* AOB amplification, respectively (Metabion, Germany), 1× Power SYBR Green PCR master mix (Applied Biosystems), and 40 ng DNA template. All PCR reactions started with an initial enzyme activation step performed at 95°C for 10 min. The subsequent thermal profile was different for each gene amplified (Table 3). The specificity of the amplification products was confirmed by melting-curve analysis and migration on 2% agarose gel. No template controls gave null or negligible values. To avoid inhibitory effects on qPCR, samples were diluted 10-fold based on a pre-experiment (data not shown). Dilution series of a plasmid with cloned *Nitrosomonas multiformis* ATCC25196 *amoA* gene (*amoA* AOB), the fosmid clone 54d9 (Leininger et al., 2006) for archaeal *amoA, Nitrobacter hamburgensis* X14 (DSMZ 10229) *nxrA* gene (*Nitrobacter*-like *nxrA)*, and the *Nitrospira* 16S rRNA gene (Accession No. FJ529918; *Nitrospira*-like 16S rRNA gene), were used to generate respective standard curves ranging from 10^1^ to 10^6^ gene copies μl^−1^ with efficiencies ranging from 94 to 98%, 98% to 100%, 93 to 98%, and 93% to 99%, respectively.

**Table 3:** **Primers and thermal profiles used for real-time PCR quantification of bacterial and archaeal *amoA*, *Nitrobacter*-like *nxrA*, and *Nitrospira*-like 16S rRNA gene**.

**Target gene**	**Primer set**	**References**	**Thermal profile**	**Cycles**	**Amplicons size (bp)**
AOB *amoA*	amoA-1F	Rotthauwe et al., 1997	94°C/60 s, 58°C/60 s, 72°C/60 s	40	500
	amoA-2R	Rotthauwe et al., 1997			
AOA *amoA*	19F	Leininger et al., 2006	94°C/45 s, 55°C/45 s, 72°C/45 s	40	624
	CrenamoA616r48x	Schauss et al., 2009			
*Nitrobacter nxrA*	F1norA	Poly et al., 2008	94°C/30 s, 55°C/30 s, 72°C/30 s	40	322
	R2norA	Wertz et al., 2008			
*Nitrospira* 16S	Nspra675f	Graham et al., 2007	94°C/30 s, 64°C/30 s, 72°C/60 s	40	71
	Nspra746r	Graham et al., 2007			

### Cloning and sequencing of archaeal *amoA* and *nitrobacter*-like *nxrA* fragments sequences and phylogenetic analysis

Prior to PCR amplification, replicates corresponding to the treatments PM and PMSDZ 1 d, 49 d and 106 d after the first manure application were pooled together to constitute one sample corresponding to one treatment at one time point.

Archaeal *amoA* and *nxrA* gene amplicons were generated by PCR using the primers described for qPCR assay (Table 3). The reaction mixture (50 μL) contained 1× PCR buffer, 1× CoralLoad concentrate, 1× Q-solution, 1 U TopTaq (Qiagen, Germany), 200 μM of each dNTP, 0.2 μM of each primer, and 30 ng template DNA. The PCR thermocycling program for *nxrA* amplification was 94°C for 3 min, followed by 35 cycles of 94°C for 30 s, 55°C for 30 s and 72°C for 1 m, and a final elongation step at 72°C for 10 min. A similar program was used for *amoA* AOA amplification with an annealing temperature of 50°C. The cloning was carried out using the TA cloning kit (Invitrogen, Germany) in accordance with the manufacturer's instructions. Thirty clones were picked randomly for each treatment and time point. Plasmids were extracted using the NucleoSpin plasmid kit (Machery-Nagel, Germany). Inserts from clones amplified with specific primers (M13 forward and M13 reverse) using the BigDye Terminator cycle sequencing kit (Applied Biosystems) were purified by ethanol precipitation. *amoA* AOA and *nxrA* fragments were sequenced using an ABI 3730 DNA analyzer (Applied Biosystems). Sequences were run through a mega BLAST search (http://blast.ncbi.nlm.nih.gov/Blast.cgi) using the nr database and were deposited in the Genbank with the accession numbers KC137376-KC137546 and KC152658-KC152839 for *amoA* and *nxrA*, respectively. For further analysis nucleotide sequences were transcribed to aminoacid sequences. These were aligned using clustal W protein alignment (Thompson et al., 1994) implemented in ARB (Ludwig et al., 2004). The nucleotide sequences were realigned according to aligned protein sequences. DNA-based maximum likelihood trees were reconstructed applying PhyML (Guindon and Gascuel, 2003) implemented in ARB. Rarefaction curves were created using Mothur for a distance of 0.02 (98% similarity level) (Schloss et al., 2009). The clustering of *amoA* AOA was done according to the method of Pester et al. (2012).

### Statistical analysis

Inorganic nitrogen and gene abundance data were analyzed by two-way analysis of variance (ANOVA) with treatment (PM, PMSDZ) and time (after the first [1 d, 7 d, 14 d, and 42 d] and the second [49 d, 56 d, 63 d, and 106 d] manure application) as independent factors. In addition, the Dunnett-T3 was used as a *post-hoc* test. Homogeneity of the variances was checked by the Levene test. The significance was set to α = 0.05. Prior to analysis, gene abundance data were ln-transformed to achieve normal distribution. Independent *T*-tests were used to test for a significant difference between the two treatments at a given time point with significance level corrected by the Šidák's equation to α = 1 − (1 − 0.05)^1/4^ = 0.013 (Šidák, 1967), as each manure application was followed by four sampling time points. Statistical tests were calculated in SPSS 11.5 (SPSS, Inc., IL, USA).

## Results

### Inorganic nitrogen

**Figure 1:**
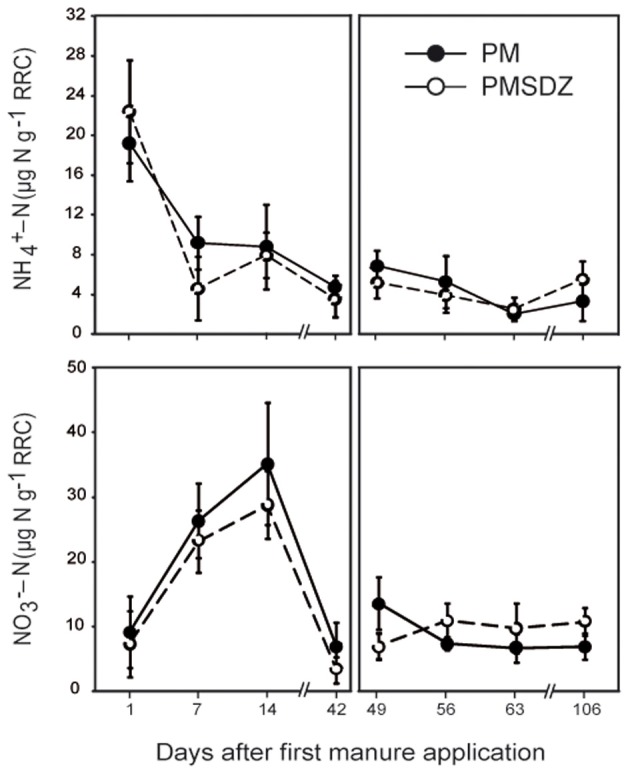
**Ammonium and nitrate concentrations in the root-rhizosphere complex (RRC) of pasture plants after addition of pig manure (PM; full circles, •) or SDZ-contaminated pig manure (PMSDZ; empty circles, ◦) 1 d, 7 d, 14 d, and 42 d after primary manure application**. Also included are measurements at 49 d, 56 d, 63 d, and 106 d post primary application, made after second manure application occurring 48 d post primary application. Error bars represent standard deviation of mean of replicate samples (*n* = 4). Abbreviations: RRC, root-rhizosphere complex.

The highest NH^+^_4_-N concentrations were measured at day 1, independent from the treatment (up to 22.8 μg NH^+^_4_-N per gram of dry weight RRC). Significant time-dependent differences were detected after the two applications of manure (Table A1). Only one week later the values dropped to below 10 μg NH^+^_4_-N g^−1^ RRC in the PM treatments and below 5 μg NH^+^_4_-N g^−1^ RRC in the PMSDZ treatments. However, differences between the two treatments were not significant. This level remained constant during the experimental period; the second manure application at day 49 did not influence NH^+^_4_-N concentrations in the RRC (Figure 1).

Time had a significant effect on NO^−^_3_-N concentrations after the first manure application (Table A1). NO^−^_3_-N concentrations peaked independently from the antibiotic treatment 14 d after the first application of manure (up to 35.4 μg NO^−^_3_-N g^−1^ RRC). Toward day 42, the values dropped sharply and were close to the detection limit in some of the replicates. Similarly to the observation regarding NH^+^_4_-N concentrations, the second manure application had no effect on the NO^−^_3_-N concentrations in the RRC independent of the treatment (Figure 1).

### Gene abundance

**Figure 2:**
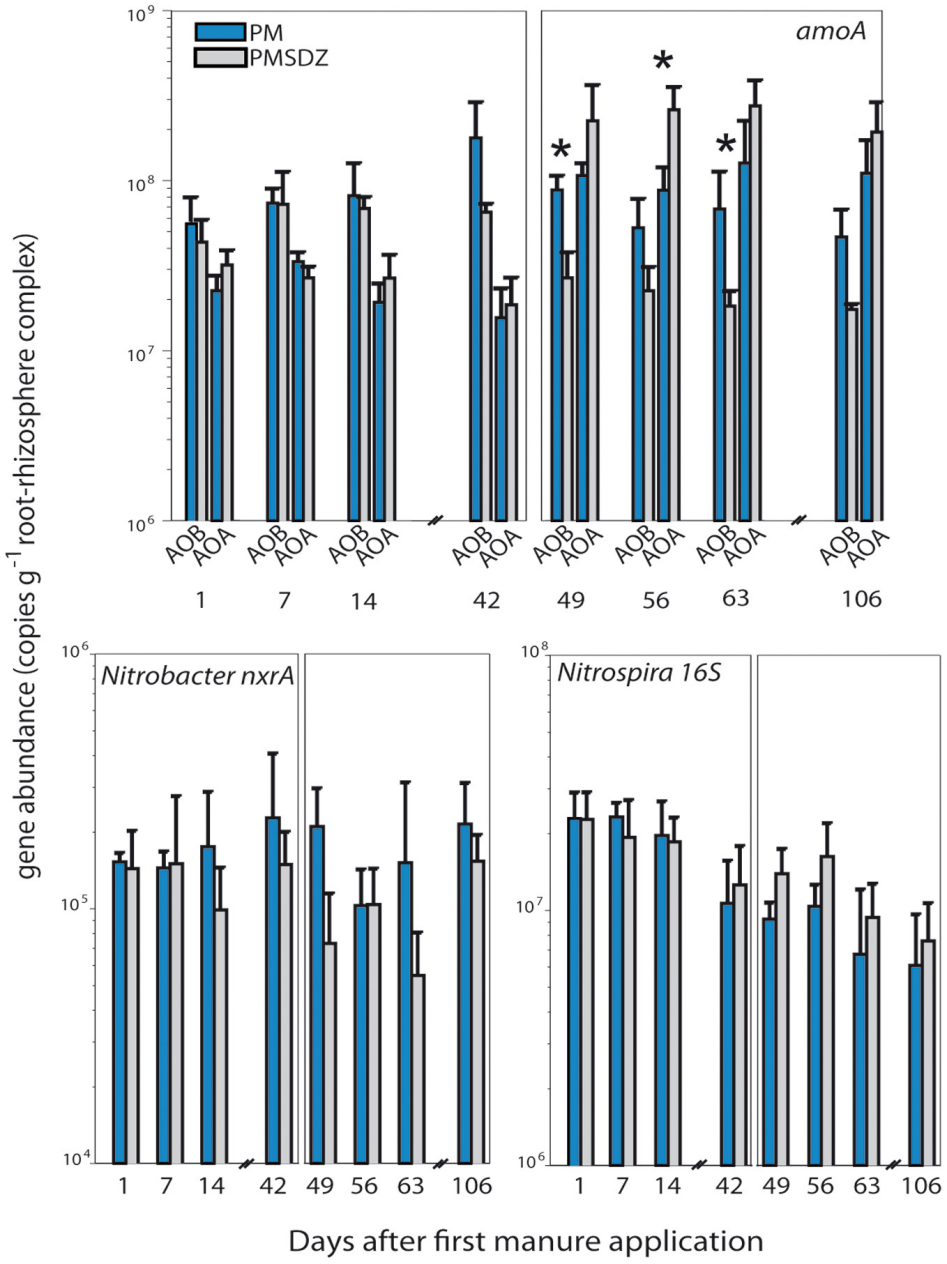
**Quantification of *amoA* (AOB and AOA), *Nitrobacter*-like nxrA and *Nitrospira*-like 16S rRNA genes in the root-rhizosphere complex (RRC) of pasture plants after addition of pig manure (PM, blue bars) or SDZ-contaminated pig manure (PMSDZ, gray bars) 1 d, 7 d, 14 d, and 42 d after primary manure application**. Also included are measurements at 49 d, 56 d, 63 d, and 106 d post primary application, made after second manure application occurring 48 d post primary application. Significant differences between the two treatments at a particular time point are indicated by asterisks (^*^) for each investigated gene. Error bars represent standard deviation of mean (*n* = 4). Abbreviations: RRC, root-rhizosphere complex.

The first application of manure did not influence *amoA* gene copy numbers for AOA and AOB 1, 7, and 14 d after application (Figure 2, Table A2). Higher copy numbers (in the range of 6.5 × 10^7^ copy numbers per gram of dry weight RRC) were measured for AOB; AOA copy numbers were at the same time points were slightly lower (in the range of 2.5 × 10^7^ copies g^−1^ RRC) resulting in an AOA:AOB ratio of 0.4. No significant influence of SDZ was visible at these time points either on AOA or AOB. However, 42 d after application, AOB *amoA* gene copy numbers in the treatments with control manure (PM) increased up to 1.8 × 10^8^ copies g^−1^ RRC, whereas in the plots where contaminated manure (PMSDZ) was applied no changes were visible compared to the earlier time points. AOA *amoA* gene abundance did not differ 42 d after application of the manure compared to the earlier time points. Therefore, at this time point AOA:AOB ratio in the PM treatment was the lowest measured (0.1). After the second manure application *amoA* AOA and AOB copy numbers in the RRC of plants from the PM treated plots were comparable, as *amoA* AOA copy numbers increased compared to earlier sampling time points (ratio AOA:AOB = 1). A clear influence of the antibiotic was visible on both AOA and AOB at all time points investigated. Copy numbers for *amoA* AOB decreased in PMSDZ plots compared to the control samples. Thus, values were in the range of 2 × 10^7^ gene copies g^−1^ RRC in PMSDZ plots compared to 7 × 10^7^ g^−1^ RRC in the plots treated with PM. In contrast AOA *amoA* gene copy numbers increased significantly in the RRC of plants in PMSDZ-treated plots compared to control plots, with gene copy numbers in the range of 3 × 10^8^ copies g^−1^ RRC, whereas copy numbers in the control treatment were approximately 1 × 10^8^ copies g^−1^ RRC. Interestingly, the described effect was stable at day 106. Consequently, AOA:AOB ratio increased up to 15 after the second application of SDZ-contaminated manure.

Copy numbers for 16S rRNA genes from *Nitrospira* ranged from 6.1 × 10^6^ to 2.3 × 10^7^ gene copies g^−1^ RRC during the experimental period (Figure 2). The highest gene copy numbers were measured after the first manure application at time point 1 d. The lowest gene copy numbers were detected at the end of the experimental period at day 106. The second manure application did not increase 16S rRNA gene copies of *Nitrospira*. Surprisingly SDZ had no significant influence on the abundance of *Nitrospira* over the experimental period. The abundance of *nxrA* genes from *Nitrobacter* were 2 orders of magnitude lower compared to 16S rRNA gene copy numbers of *Nitrospira*. Over all time points, none of the two manure applications changed the *nxrA* gene abundance significantly. Copy numbers ranged between 1.0 × 10^5^ and 2.3 × 10^5^ copies g^−1^ RRC in PM-treated plots. We observed a tendency for reduced gene copies 49 d after the first application of the PMSDZ compared to the control PM treatment group (*P* = 0.030).

### Diversity of AOA *amoA* and *nitrobacter*-like *nxrA* genes

**Figure 3:**
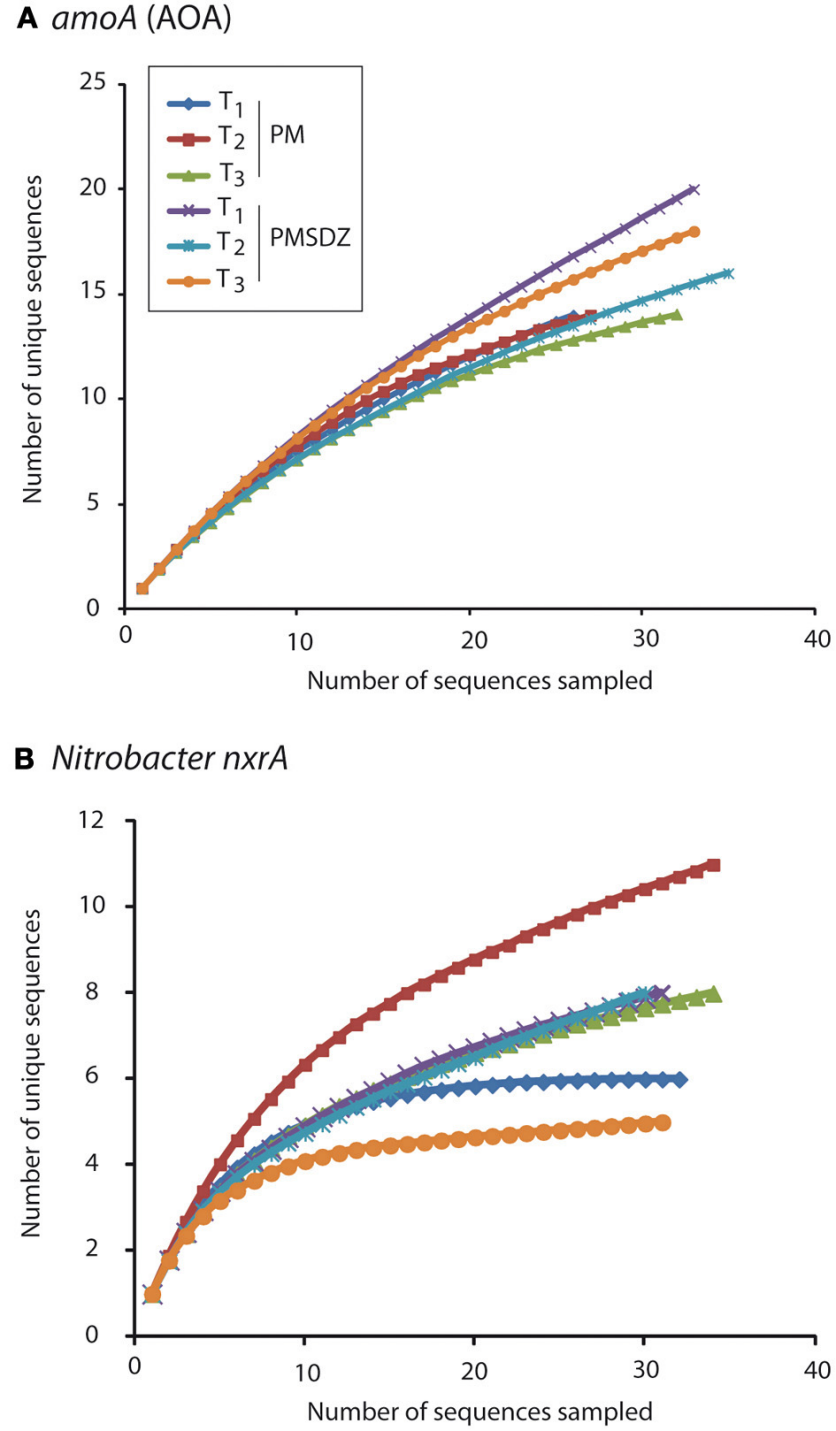
**Rarefaction curves for *amoA* AOA (A) and *Nitrobacter*-like *nxrA* (B) sequences obtained at three different time points (1 d, T_1_; 49 d, T_2_; and 106 d, T_3_) after application of pig manure (PM) or SDZ-contaminated pig manure (PMSDZ)**. The curves express the number of OTUs as a function of the number of the sequences sampled in each library.

**Figure 4:**
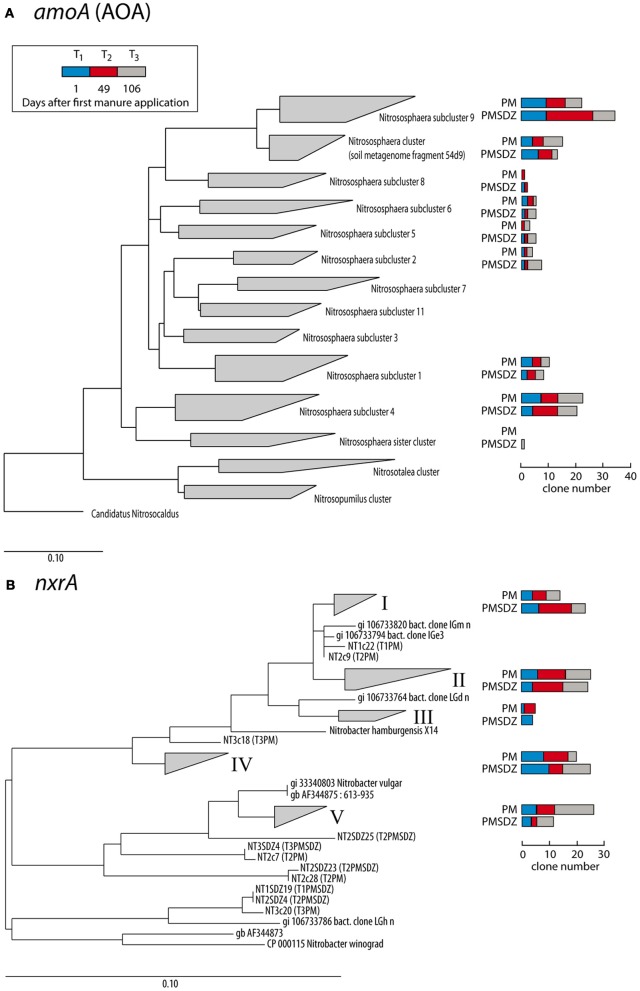
**Maximum likelihood trees of partial *amoA* AOA (A) and *Nitrobacter*-like *nxrA* (B) nucleic acid sequences (624 and 322 bp, respectively) obtained at three different time points (1 d, blue bars; 49 d, red bars; and 106 d, gray bars) after application of pig manure (PM) or SDZ-contaminated pig manure (PMSDZ) and reference sequences**. AOA *amoA*-clustering was performed according to Pester et al. (2012). Ungrouped trees are available in Figures A1 and A2.

**Figure A1:**
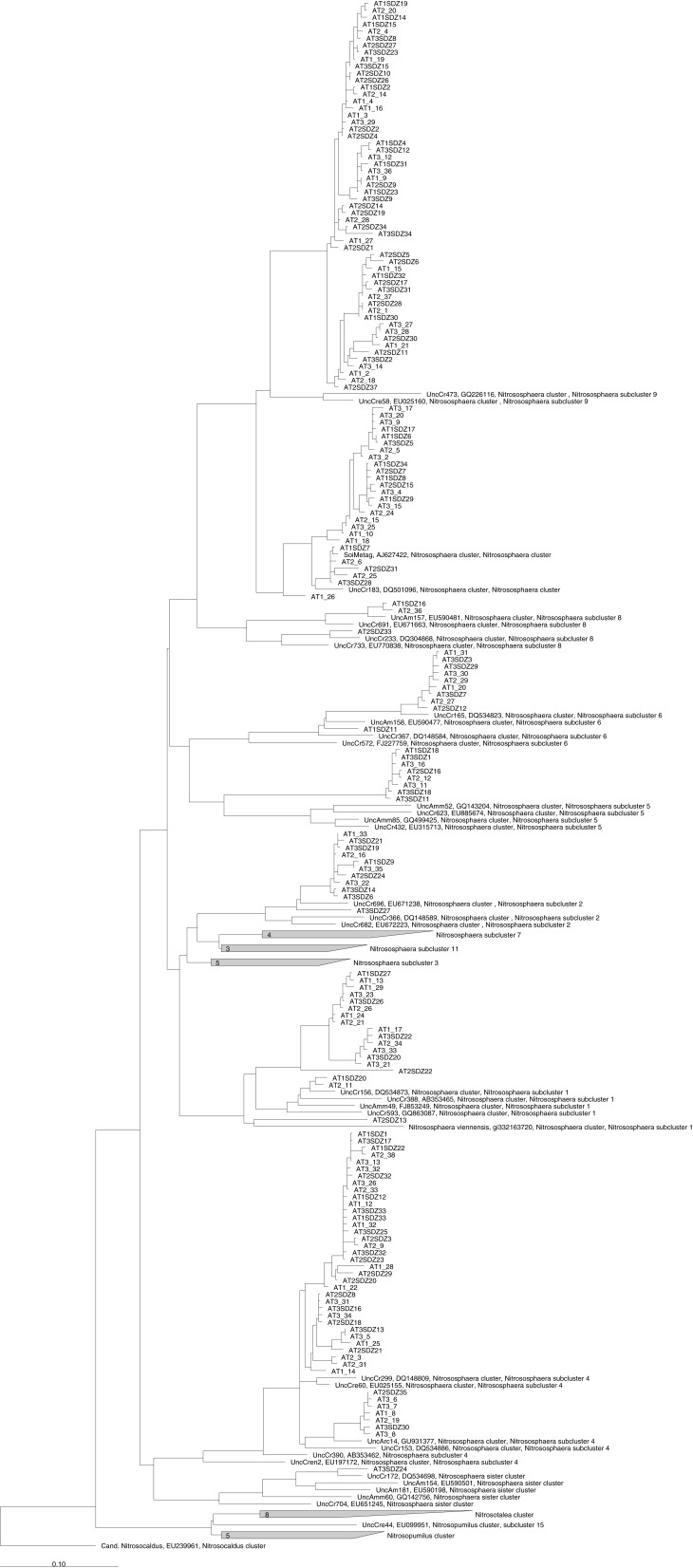
**Phylogenetic analysis of partial *amoA* nucleic acid sequences (624 bp).** This figure represents the ungrouped phylogenetic tree found in Figure 4A. Clone names include indications on the sampling time point (T1, T2, T3) as well as the treatment (SDZ for PMSDZ).

The clones per library sequenced for each treatment did not account for the total diversity of AOA *amoA* OTUs present in the samples as indicated by the rarefaction curves (Figure 3). The total number of OTUs for *amoA* AOA varied from 12 to 14 in the PM-treated plots, independent from the time point of sampling. In contrast in the PMSDZ treatment, the number of OTUs decreased between day 1 and day 49, and thereafter increased between day 49 and day 106 after the first application. Overall, 85% of the sequences sampled were contained in *Nitrosphaera* subclusters 1, 4, and 9; and in the *Nitrosphaera* cluster (soil metagenome fragment 54d9). *Nitrosphaera* subclusters 1 and 4 were represented by sequences from all treatments and time points. However, in *Nitrosphaera* subcluster 9, the abundance of sequences from the treatment PMSDZ one day after the second manure application (day 49) was higher compared to PM. In contrast, in the *Nitrosphaera* cluster (soil metagenome fragment 54d9), at the 106-d point, the abundance of sequences from the control treatment was higher compared to PMSDZ (Figures 4, A1).

**Figure A2:**
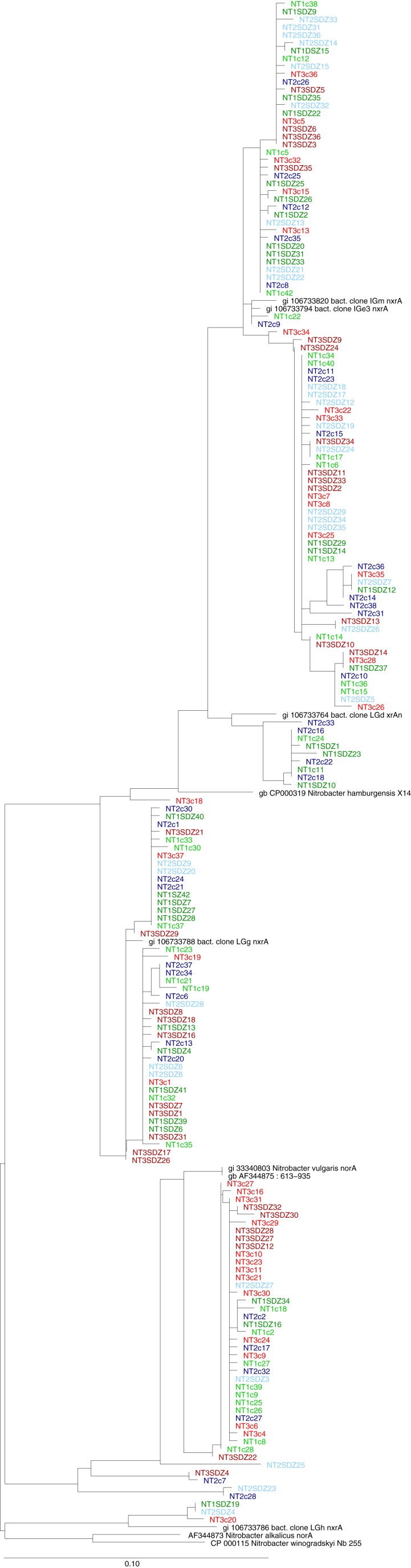
**Phylogenetic analysis of partial *nxrA* nucleic acid sequences (322 bp).** This figure represents the ungrouped phylogenetic tree found in Figure 4B. Clone names include indications on the sampling time point (T1, T2, T3) as well as the treatment (SDZ for PMSDZ).

The number of different OTUs for *nrxA* was lower compared to AOA *amoA*. Therefore, the investigated 30 clones per library largely reflected the diversity present in the samples and the collector's curves indicated saturation. The total number of OTUs for *nrxA* ranged between 4 and 11. However, the observed diversity pattern followed the opposite trend compared to AOA *amoA*. The highest number of OTUs was observed in the treatments with control manure one day after the second application (day 49). The lowest number of OTUs was observed at the last sampling time point (day 106) in the PMSDZ-treated plots. However, the number of OTUs was already reduced at this time point also in the control samples compared to day 49 (Figure 3). Four major clusters were detected, containing 89% of the total *nxrA* sequences sampled (I, II, IV, and V; Figures 4, A2). Each individual cluster was represented by sequences from both treatments and all three time points. In the treatment where contaminated manure was added, the relative abundance of sequences 1 d after the second application of the manure was higher in cluster I vs. the control treatment. A similar trend was observed for the last sampling time point (day 106) for cluster IV. An opposite trend was observed in cluster V, where a higher number of sequences was found from the control treatment at the last sampling time point (day 106).

## Discussion

### Effects on ammonia oxidizers

The antibiotic tended to abolish the increase of the abundance of AOB after 42 d in response to the manure application in RRC of the pasture plants (*P* = 0.091) (Figure 2). Similar results have been reported in analyses of bulk soil (Schauss et al., 2009) and in the rhizosphere of agricultural crops (Ollivier et al., 2010). These results demonstrate that SDZ inhibits the growth of AOB populations (considering gene abundance as proxy). The response of bacterial and archaeal ammonia oxidizers, respectively, differed one day after the second SDZ-contaminated manure application. From this time point onward, the *amoA* AOB and AOA gene abundance patterns were decreased and increased, respectively (Figure 2). Although it has been already shown that AOA were impacted to a lower extent by SDZ than AOB in greenhouse experiments (Schauss et al., 2009; Ollivier et al., 2010), in this study we observed for the first time a significant increase in *amoA* AOA gene copies (*P* = 0.005 at day 56) while *amoA* AOB gene copies were significantly decreased (*P* = 0.005 and *P* = 0.013 at day 49 and 63, respectively) with PMSDZ treatment, confirming the potential occurrence of functional redundancy between the two communities under antibiotic stress (Schauss et al., 2009). However, caution must be taken when relating significant impacts of the antibiotic on the gene level to associated functions. Indeed, whereas SDZ has been shown to affect corresponding gene expression to a lower extent, Ollivier et al. (2010) suggested that microbial subpopulations intrinsically able to cope with the antibiotic stressor could take advantage of the altered competitive environment and maintain the function.

Possibly, the reduced susceptibility of AOA to PMSDZ can be explained by a shift in the AOA diversity toward more SDZ tolerant phylotypes over time. In this respect *Nitrosphaera* subcluster 9 might be of interest: whereas the number of clones which could be assigned to this cluster did not differ between both treatments 1 d after the first application of manure, 1 d after the second application of manure, an increased number of clones derived from PCR products obtained from the PMSDZ-treated soil samples showed high similarities to this subcluster (Figure 4). However, it should be noted that the development of antibiotic resistance in AOA has not been described so far, as the first cultures of soil AOA have been isolated only recently (Jung et al., 2011; Tourna et al., 2011; Kim et al., 2012).

### Effects on nitrite oxidizers

Because of the broad-spectrum nature of the SDZ antibiotic, we hypothesized that *Nitrospira*- and *Nitrobacter*-like nitrite oxidizers are both inhibited by the application of the contaminated manure. However, parallel to the inhibitory effects directly affecting the functional communities investigated, e.g., related to their respective activity status and related susceptibility (Lewis, 2007; Ollivier et al., 2010) or to their abilities to regulate their internal pH, which affect the accumulation and speciation of the SDZ in the cells (Tappe et al., 2008; Zarfl et al., 2008), effects that can be considered as indirect and potentially related to other nutrient cycles than nitrogen turnover, food web development, susceptibility to phages or changes in soil physicochemical properties (e.g., changed redox conditions due to an overall reduced activity of the soil microflora in response to the antibiotic) may explain their response to the antibiotic stress. While niche differentiation and competition is known to influence the composition of functional microbial communities, the components of the nitrite-oxidizing communities investigated, respectively, in this study have been reported to possess different substrate affinities and therefore are adapted to distinct N availabilities (Schramm et al., 1999). Thus, it has been suggested that *Nitrobacter*-like NOB bacteria are r-strategists with higher growth rate/specific activity and lower affinity for nitrite and oxygen, whereas *Nitrospira*-like NOB are K-strategists with a higher substrate affinity (Schramm et al., 1999; Attard et al., 2010). However, Maixner et al. (2006) have shown that the nitrite concentration influences the structure of *Nitrospira*-like bacterial communities, and assumed that sublineages may occupy different positions on a scale reaching from K- to r-strategists within the genus *Nitrospira*. In the RRC of the pasture plants from 42 days after the first manure application, the reduction of AOB abundance and activity may have resulted in lower nitrite availability and consequently favorable conditions for *Nitrospira*-like NOB compared to *Nitrobacter*-like NOB, explaining the reduction of *Nitrobacter*-like *nxrA* abundance (*P* = 0.030) and the increase of *Nitrospira* 16S rRNA gene abundance (*P* = 0.036) at day 49 with PMSDZ treatment. Moreover, whereas nitrite reductase homologues have been reported in AOA (Bartossek et al., 2010), the increase in abundance of AOA could have further decreased nitrite availability, and consequently collapsed the niche of *Nitrobacter*-like NOB. However, this would require changes in the ecophysiology of AOA (from autotrophic to heterotrophic lifestyle) and shifts in the redox potential at the present microsites toward oxygen limitation. Furthermore, a putative disturbance of the carbon cycle under the antibiotic treatment could have also indirectly influenced the abundance of different *Nitrospira* sublineages as some *Nitrospira*-like bacteria are mixotrophic (Daims et al., 2001).

A community shift due to SDZ treatment was observed for *Nitrobacter*-like NOB at the 49-d point in cluster I. Moreover, the antibiotic treatment had a long-lasting effect on these communities as differences between the two communities, specifically in the relative abundance for cluster IV and V, were observed 106 d after the first application of manure, as well as a decrease in diversity (Figure 3). Therefore, whereas SDZ-resistant phylotypes might have developed in response to the antibiotic stress, there was no recovery of microbial diversity after the PMSDZ treatment during the experimental period. Possibly, critical nitrifying consortia might have been disturbed owing to the antibiotic stress, which may imply negative effects on the ability of the soil to respond to future disturbances (Ives et al., 2000; McCann, 2000). Furthermore, the observed changes in *Nitrobacter*-like NOB diversity could be indirectly explained by the increased AOA abundance, as mentioned above.

### Consequences for nitrification and N turnover

This field experiment revealed that the application of manure contaminated with the antibiotic SDZ has a lasting effect on the abundance and diversity of nitrifying microbial communities. However, although SDZ impaired the growth of certain microbial populations, there was no significant effect of the PMSDZ treatment on the concentrations of nitrate and ammonium in the rhizosphere of the plants composing the pasture. Differences in tolerance between the different microbial functional communities, or the development of tolerant populations (Heuer et al., 2011) could contribute to functional redundancy of ammonia-oxidizing microbes (Schauss et al., 2009) and NOB, and therefore to the stability of nitrification pattern in the rhizosphere. However, the studied system was not shown to recover in terms of *nxrA* diversity after the PMSDZ treatment during the experimental period, which could indicate that mostly nitrite oxidation by NOB might be affected by the application of SDZ. Possibly, alternative pathways like nitrite reduction might be favored under these conditions; as many soil microbes are able to use nitrite as terminal electron acceptors under anaerobic conditions, the extent of the SDZ-contamination effects on denitrification may be reduced.

### Potential pitfalls of this study

On the first glance, one might speculate that ammonia oxidation is more resilient and influenced to a limited extent by the antibiotic due to functional redundancy of AOA and AOB. However, this study only gives data on the abundance of genes (genetic potential) and does not take into account enzymes, which drive the particular process. Therefore, additional proteomic based approaches might be needed to clarify if indeed functional redundancy exists and AOA actually takes over the capability to oxidize ammonia from AOB. Furthermore, while AOB gene abundance might be at least a proxy for ammonia oxidation activity (as an increase in abundance of AOB requires the oxidation of ammonia due to their purely autotrophic lifestyle), this is different for AOA, where also heterotrophic growth, independent from ammonia oxidation, could be proven by using urea as alternative nitrogen source (Tourna et al., 2011). Therefore, in the case of AOA, changes in the abundance are not necessarily linked to ammonia oxidation.

Another issue of concern for data interpretation is the difference in quality between the manure produced by animals that were treated with antibiotics and control animals. It is well known that antibiotics change the physiology of mammals and therefore differences in the composition of the manure would not be surprising (Antunes et al., 2011). Thus, it cannot be completely excluded that some of the observed differences in response to the second application of the antibiotic are related to the slight differences in the nutrient composition of the manure. However, spiking the manure with SDZ would also result in a biased picture, as important metabolites of SDZ are only formed during the passage through the body of the pigs (Lamshöft et al., 2007).

## Outlook

In this study, the rhizosphere as an important soil compartment has been investigated. However, the type of response of the targeted functional communities might be differing in other soil compartments, e.g., the drilosphere where microbes form different types of consortia and produce extracellular polymeric substances (EPS) in different amounts (Brown et al., 2000), which might reduce the effects of the antibiotic compared to the rhizosphere. Of course, the soil type and the environmental conditions present at the site may also highly influence the response pattern of the microflora toward antibiotics, as well as the land use history of the soil (essentially the repeated application of manure contaminated with antibiotics) in the years before the experiment started (Berg and Smalla, 2009; Heuer et al., 2011; Rosendahl et al., 2011). These aspects may be considered in a study where attempts are made to generalize the obtained data. Moreover, whereas plant diversity and species composition are known to influence the magnitude and the stability of ecosystem processes over time, as well as the size and composition of associated microbial communities (Hooper and Vitousek, 1997; Kowalchuk et al., 2002; Steenwerth et al., 2002; Johnson et al., 2003; Balvanera et al., 2006; Millard and Singh, 2010), the question of how interactive effects between the antibiotic and plant diversity might influence microbial communities needs to be investigated into further experiments. Most important, however, will be the use of proteomic-based approaches to link the genetic potential of a soil to turnover processes in order to unveil the secrets of “functional redundancy.”

### Conflict of interest statement

The authors declare that the research was conducted in the absence of any commercial or financial relationships that could be construed as a potential conflict of interest.

## Appendix

Table A1 **Statistical evaluation of ammonium-N and nitrate-N concentrations in the root-rhizosphere complex by two-way ANOVA**.

**Table:**

**Factor**	***P***	
	**NH^+^_4_-N**	**NO^−^_3_-N**
**AFTER THE FIRST MANURE APPLICATION (1 D, 7 D, 14 D, 42 D)**				
Treatment	0.946	0.128
Time	**0.000**	**0.000**
Treatment × Time	0.898	0.919
**AFTER THE SECOND MANURE APPLICATION (49 D, 56 D, 63 D, 106 D)**				
Treatment	0.900	0.371
Time	**0.015**	0.551
Treatment × Time	0.273	**0.007**

**Table:**

	**Time**	**Time**	**Significance**	
			**NH^+^_4_-N**	**NO^−^_3_-N**
Dunnett-T3 *post-hoc* test		7 d	**0.007**	**0.001**
	1 d	14 d	**0.001**	**0.002**
		42 d	**0.000**	0.728
		1 d	**0.007**	**0.001**
	7 d	14 d	1.000	0.430
		42 d	0.274	**0.000**
		1 d	**0.001**	**0.002**
	14 d	7 d	1.000	0.430
		42 d	0.092	**0.001**
		1 d	**0.000**	0.728
	42 d	7 d	0.274	**0.000**
		14 d	0.092	**0.001**
		56 d	0.722	0.992
	49 d	63 d	**0.008**	0.909
		106 d	0.545	0.975
		49 d	0.722	0.992
	56 d	63 d	0.199	0.991
		106 d	1.000	1.000
		49 d	**0.008**	0.909
	63 d	56 d	0.199	0.991
		106 d	0.405	0.999
		49 d	0.545	0.975
	106 d	56 d	1.000	1.000
		63 d	0.405	0.999

*Bold characters indicate significant effects of treatment, time, and the interaction between treatment and time (Treatment × Time), respectively (P < 0.05)*.

Table A2 **Statistical evaluation of gene abundances by two-way ANOVA**.

**Table:**

**Factor**			***P***			
			***amoA AOB***	***amoA AOB***	***Nitrobacter nxrA***	***Nitrospira 16S***
**AFTER THE FIRST MANURE APPLICATION (1 D, 7 D, 14 D, 42 D)**						
Treatment			0.088	0.256	0.192	0.910
Time			**0.024**	**0.009**	0.606	**0.004**
Treatment × Time			0.291	0.408	0.934	0.722
**AFTER THE SECOND MANURE APPLICATION (49 D, 56 D, 63 D, 106 D)**						
Treatment			**0.000**	**0.000**	**0.029**	**0.030**
Time			0.107	0.958	**0.049**	**0.026**
Treatment × Time			0.727	0.719	0.333	0.962

**Table:**

	**Time**	**Time**	**Significance**			
			***amoA AOB***	***amoA AOA***	***Nitrobacter nxrA***	***Nitrospira 16S***
Dunnett-T3 *post-hoc* test		7 d	0.326	0.965	0.999	0.990
	1 d	14 d	0.293	0.727	0.951	0.709
		42 d	0.089	0.107	0.983	**0.023**
		1 d	0.326	0.965	0.999	0.990
	7 d	14 d	1.000	0.280	0.997	0.982
		42 d	0.672	**0.041**	0.936	0.054
		1 d	0.293	0.727	0.951	0.709
	14 d	7 d	1.000	0.280	0.997	0.982
		42 d	0.727	0.601	0.774	0.133
		1 d	0.089	0.107	0.983	**0.023**
	42 d	7 d	0.672	**0.041**	0.936	0.054
		14 d	0.727	0.601	0.774	0.133
		56 d	0.832	1.000	0.997	0.940
	49 d	63 d	0.904	1.000	0.800	0.431
		106 d	0.524	1.000	0.686	0.098
		49 d	0.832	1.000	0.997	0.940
	56 d	63 d	1.000	1.000	0.886	0.248
		106 d	0.997	1.000	0.084	**0.044**
		49 d	0.904	1.000	0.800	0.431
	63 d	56 d	1.000	1.000	0.886	0.248
		106 d	0.996	0.999	0.145	1.000
		49 d	0.524	1.000	0.686	0.098
	106 d	56 d	0.997	1.000	0.084	**0.044**
		63 d	0.996	0.999	0.145	1.000

*Bold characters indicate significant effects of treatment, time, and the interaction between treatment and time (Treatment × Time), respectively (P < 0.05)*.
